# Whole Genome Transcriptome Analysis of the Association between Obesity and Triple-Negative Breast Cancer in Caucasian Women

**DOI:** 10.3390/ijerph15112338

**Published:** 2018-10-23

**Authors:** Tarun K. K. Mamidi, Jiande Wu, Paul B. Tchounwou, Lucio Miele, Chindo Hicks

**Affiliations:** 1 Department of Genetics, Louisiana State University Health Sciences Center, School of Medicine, 533 Bolivar Street, New Orleans, LA 70112, USA; tmamid@lsuhsc.edu (T.K.K.M.); jwu2@lsuhsc.edu (J.W.); lmiele@lsuhsc.edu (L.M.); 2 NIH/NIMHD RCMI Center for Environmental Health, Jackson State University, 1400 Lynch Street, Box 18750, Jackson, MS 39217, USA; paul.b.tchounwou@jsums.edu

**Keywords:** gene expression, obesity, triple-negative breast cancer

## Abstract

Background: Triple-negative breast cancer (TNBC) is the most aggressive form of breast cancer, with poor outcomes. The molecular basis of TNBC remains poorly understood. The objective of this exploratory study was to investigate the association between obesity and TNBC in premenopausal and postmenopausal Caucasian women using transcription profiling. Methods: We compared gene expression levels of tumor samples drawn from normal weight, overweight, and obese pre and postmenopausal women diagnosed with TNBC. We performed hierarchical clustering to assess similarity in patterns of gene expression profiles, and conducted network and pathway analysis to identify molecular networks and biological pathways. Results: We discovered gene signatures distinguishing normal weight from obese, normal weight from overweight, and overweight from obese individuals in both premenopausal and postmenopausal women. The analysis revealed molecular networks and biological pathways associating obesity with TNBC. The discovered pathways included the unfolded protein response, endoplasmic reticulum stress, B cell receptor, and autophagy signaling pathways in obese premenopausal women; and the integrin, axonal guidance, ERK/MAPK (extracellular-signal-regulated kinase/mitogen activated protein kinase) and glutathione biosynthesis signaling pathways in obese postmenopausal women. Conclusions: The results suggest that both overweight and obese status are associated with TNBC, highlighting the need for conformation of these results in independent studies.

## 1. Introduction

Triple negative breast cancer (TNBC) represents breast cancers which lack expression of the estrogen receptor (ER) and progesterone receptor (PR) and show lack of amplification of the human epidermal growth factor receptor 2 (*HER2*) gene [1]. TNBC is a heterogeneous disease with a complex etiology. It is the most aggressive form of breast cancer, with very poor clinical outcomes. Although TNBC represents only 15% of all breast cancers, it accounts for 25% of all breast cancer-related deaths [1,2]. Women with TNBC have a high frequency of metastasis to the lung, liver, and brain, and the survival rate is poor [3]. Even more concerning is that the median survival rate for women with metastatic TNBC is less than one year [3]. To date, there are no effective targeted therapies, and chemotherapy remains the only effective therapeutic modality [1,2,3]. Therefore, there is a pressing need to understand the biological factors and pathways that drive these tumors and discover molecular markers for the development of targeted therapies.

Over the last decade, there has been growing interest in investigating the association between obesity and TNBC. This has been driven in part by the realization that modifying factors such as socio-economic status and lifestyle may be associated with the disease [4,5,6,7]. However, the results have been inconsistent and in some cases contradictory. Several epidemiologic studies have reported the association of overweight and obese status with TNBC [8,9]. Overweight status and obesity have also been associated with overall survival (OS) rate and disease-free survival rate (DFS) [10,11,12,13,14,15,16]. However, other epidemiologic studies did not find the association between obesity and or overweight with TNBC [17,18,19]. These seemingly contradictory results underscore the need for further research in this area.

In clinical practice, studies have shown that obesity is an independent prognostic factor of decreased pathological complete response to neoadjuvant chemotherapy in breast cancer patients [20,21]. A recent study on TNBC patients treated with neoadjuvant chemotherapy identified body mass index (BMI) and menopausal status as two promising prognostic factors [22]. However, the molecular mechanisms associating BMI with TNBC in premenopausal and postmenopausal women are poorly understood. Given the expanding obesity epidemic and the poor prognosis of the TNBC tumors, discovery of molecular markers associated with modifiable risk factors such as obesity may facilitate the development of novel prevention strategies and the realization of precision prevention. The objective of this exploratory study was to investigate the association of obesity and/or overweight status with TNBC in premenopausal and postmenopausal Caucasian women using transcription profiling, and to discover molecular networks and biological pathways associating obesity with TNBC. Our working hypothesis is that genomic alterations in overweight and obese premenopausal and postmenopausal women are associated with TNBC and that these genomic alterations affect entire molecular networks and biological pathways driving the disease phenotypes.

## 2. Material and Methods

### 2.1. Research Design and Source of Gene Expression Data

We used publicly available gene expression data generated using tumor samples from premenopausal and postmenopausal Caucasian women diagnosed with TNBC. The data set was downloaded from the Gene Expression Omnibus (GEO) under accession number GSE76124 [23]. The experimental procedures and methods of sample processing have been fully described by the data originators [23]. Here we provide a short but detailed summary of the data and the characteristics of the data used in this study.

The data set involved a total of 198 TNBC tumors samples collected at Baylor College of Medicine (BCM, Houston, TX, USA) with confirmed diagnosis. The samples were fresh frozen. The samples included four subtypes of TNBC defined as basal-like immune-activated (BLIA), basal-like immunosuppressed (BLIS), luminal androgen receptor (LAR), and mesenchymal (MES) and were consistent with TNBC subtype classification [23,24,25]. The tumor samples included clinical information including age, menopausal status, histology, stage, tumor grade, body mass index (BMI), and tumor size. No treatment or outcome data were available for these tumors [23]. Cellularity, histology, and IHC (immunohistochemical) ER, PR, and HER2 were assessed by breast cancer pathologists. Only tumors exhibiting >50% tumor cellularity were used.

The World Health Organization (WHO) and the Center for Disease Control and Prevention (CDC) of the United States use BMI defined as an index of weight-for-height to classify individual adults as underweight, normal weight, overweight, and obese [26,27]. Consistent with the WHO and CDC classification, in this study we used gene expression data with measurements of BMI from individual patients representing normal weight, overweight and obese for premenopausal and postmenopausal Caucasian women diagnosed with TNBC. From the original data set of 198 patients, we removed 50 individuals without menopausal status and or measurements of BMI. The final data set included a total of 148 patients distributed according to menopausal status and classified by BMI consistent with WHO and CDC classification criteria. The distribution of the 54 patients with premenopausal status was: normal weight (BMI ≤ 24.99; *n* = 21), overweight (BMI = 25–29.99; *n* = 21) and obese (BMI ≥ 30; *n* = 12). Similarly, the distribution of the 94 patients with postmenopausal status was: normal weight (BMI ≤ 4.99; *n* = 25), overweight (BMI = 25–29.99; *n* = 31), and obese (BMI ≥ 30; *n* = 38). The data set was generated using the Affymetrix platform using the Human GeneChip U133Plus 2.0 which contains 54,614 probe sets). Expression values were calculated using the robust multi-array average (RMA) algorithm as implemented in the Affymetrix platform. All the expression values ware on a log scale (log2).

### 2.2. Data Analysis

**Figure 1:**
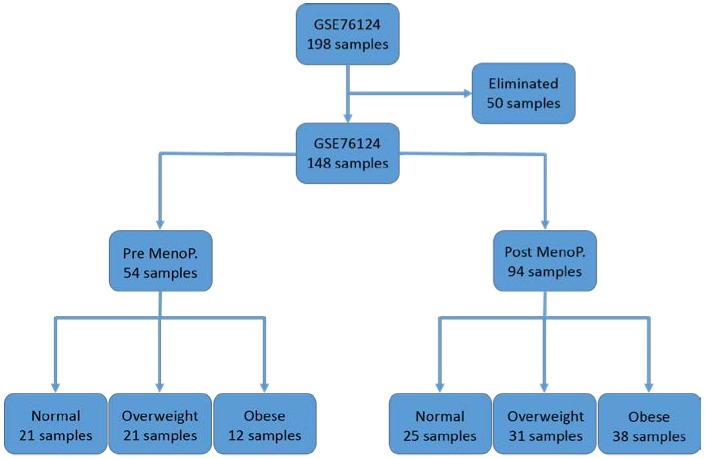
Distribution of the samples used in the analysis by menopausal status and body mass index (BMI). Note: 50 individual patients with missing information on menopausal status and BMI were excluded from the analysis.

Following processing of the data by menopausal status and BMI the overall gene expression data set was partitioned into patient groups in preparation. The overall design and data analysis workflow are presented in Figure 1.

We performed supervised analysis comparing gene expression levels among and between patient groups. We used analysis of variance (ANOVA) to compare gene expression levels among the three patient groups: normal weight, overweight and obese by menopausal status. We performed supervised analysis using a *t*-test to compare gene expression levels between patient groups (normal weight versus overweight, normal weight versus obese and overweight versus obese), separately in premenopausal and postmenopausal women using Pomelo II Software package [28]. Due to relatively small sample sizes for each patient group, we did not partition the data set into test and validation sets as such an approach would lead to bias resulting from sampling errors. To address this issue, we used the leave-one-out cross-validation procedure as our prediction and validation model to identify genes with predictive power [29]. This approach has been used successfully in gene expression data analysis to eliminate bias [29]. We used the false discovery rate (FDR) procedure to correct for multiple hypothesis testing [30]. Genes were ranked based on the *p*-values and the FDR, and highly significantly differentially expressed genes were selected for each comparison.

We performed unsupervised analysis using hierarchal clustering based on complete linkage model using the Pearson correlation coefficient as the measure of distance between pairs of genes. Prior to clustering, gene expression data was normalized using the median normalization, standardized and centered [31]. Hierarchical clustering was performed using Morpheus software [32]. We performed network and pathways analysis using Ingenuity Pathway Analysis (IPA) software [33]. Using IPA, the most highly significantly differentially expressed genes distinguishing patients with normal weight from obese patients in premenopausal and postmenopausal women were mapped onto networks and canonical pathways. The probability scores and the log *P*-values were calculated to assess the likelihood and reliability of correctly assigning the genes to the correct molecular networks and biological pathways. A false discovery rate was used to correct for multiple hypothesis testing in pathway analysis. The predicted molecular networks and biological pathways were ranked based on z-scores and log *P*-values; respectively as implemented in IPA. Gene ontology (GO) [34] analysis, as implemented in IPA, was performed on the sets of differentially expressed genes to characterize the functional relationships among sets of genes associating overweight and obesity with TNBC and to identify the molecular functions, biological processes and cellular components in which the discovered genes are involved.

## 3. Results

### 3.1. Differences in Gene Expression Levels among Patient Groups

To identify differentially expressed genes and assess variation in patterns of gene expression levels among the three patient groups, we performed analysis of variance by menopausal status. We hypothesized that the levels of gene expression differ and vary among patient groups in premenopausal and postmenopausal women. The analysis revealed significant differences in gene expression levels among patient groups. Comparison of gene expression levels among patient groups in premenopausal women revealed a signature of 1034 significantly (*p* < 0.05) differentially expressed genes, of which 242 genes were highly significantly (*p* < 0.01) differentially expressed. Among the most highly significantly (*p* < 0.001) differentially expressed genes were *CD84*, *DUXAP8*, *NPC2*, *MAGEA5*, *PAWR*, *SNX29*, *IFNGR1*, *PRKXP1*, *WIPF1*, *ABCG1*, *DPY19L1*, *MGAT4A*, *KYNU*, *RNASET2*, *COX10-AS1*, *GPRIN3*, *MMD2*, *TMED10*, *FLVCR2*, *GABBR1*, *RPL32P3*, *RAPGEF1*, and *LYST*.

Comparison of gene expression levels among patient groups within postmenopausal women produced a signature of 1551 significantly (*p* < 0.05) differentially expressed genes, of which 376 genes were highly significantly (*p* < 0.01) differentially expressed. The most highly significantly (*p* < 0.001) differentially expressed genes were *IL4R*, *TAGLN2*, *ZNF92*, *MSX1*, *EPHA2*, *SERPINE1*, *PANX1*, *PDPN*, *KEAP1*, *STK10*, *KLF9*, *JRK*, *PLK3*, *ZNF138*, *PLEC*, *FPR1*, *CR1*, *ZNF85*, *TNC*, *SLC2A3*, *ANGPT2*, *MESDC1*, *ZEB2*, *CSGALNACT1*, *MUT*, *YWHAZ*, *ZNF140*, *PRKACA*, *MVP*, *COPS8*, *SEC23A*, *NNMT*, *YWHAH*, *SRSF7*, *CD163*, *HAS2*, *SCG2*, *LRRFIP1*, *PTPRE*, *EHD4*, *ZNF736*, *LOC101927523*, *DUSP1*, *PLP2*, *TWIST2*, *WWTR1-AS1*, *GRIA3*, *SEC62*, *IL6*, *KANSL1L*, *SCARF1*, *PROM2*, *RAPGEF2*, *BCAR3*, *OSMR*, *SMAD5*, and *SPPL3*.

There was no overlap between the two sets of highly significantly differentially expressed genes in premenopausal and postmenopausal women, suggesting that molecular perturbation in premenopausal and postmenopausal may be regulated by different molecular mechanisms. As expected, there was significant variation in gene expression levels among patient groups in both premenopausal and postmenopausal women. A list of all the significantly differentially expressed genes among patient groups by menopausal status is presented in Table SA for premenopausal women and Table SB for postmenopausal women in the supplementary data to this report.

### 3.2. Association of Overweight and Obesity with TNBC in Premenopausal Women

To address the hypothesis that overweight or obesity are associated with TNBC in premenopausal women, we performed subclass mapping comparing gene expression levels between normal weight and obese patients and between normal weight and overweight patients. We sought to discover gene signatures distinguishing individuals with normal weight from those who are either obese or overweight.

Comparison of gene expression levels between normal and overweight patients revealed a signature of 1120 significantly (*p* < 0.05) differentially expressed genes. Among them, was a signature of 219 highly significantly (*p* < 0.01) differentially expressed genes. A list of 32 most highly significantly (*p* < 0.001) differentially expressed genes distinguishing normal weight from overweight individuals is presented in Table 1. A complete list of the significantly differentially expressed genes distinguishing patients with normal weight from overweight individuals is presented in Table S1 provided in the supplementary data to this report.

The analysis comparing gene expression levels between normal and obese individuals produced a signature of 1218 significantly (*p* < 0.05) differentially expressed genes. The signature included a set of 299 highly (*p* < 0.01) significantly differentially expressed genes. Table 1 shows a list of the 32 most highly significantly (*p* < 0.001) differentially expressed genes. A least of all the significantly differentially expressed genes distinguishing patients with normal weight from obese individuals is presented in Table S1 provided as supplementary data to this report.

To address the hypothesis that molecular perturbation in overweight patients significantly differs from obese patients, we compared gene expression levels between the two patient groups. The analysis revealed a signature of 635 significantly differentially expressed genes at a nominal *p*-value (*p* < 0.05). A subset of 92 genes were highly significantly (*p* < 0.01) differentially expressed.

There was a small overlap between genes associating overweight with TNBC and those associating obesity with TNBC, suggesting that overweight and obesity may be regulated by different biological mechanisms in premenopausal women. A list of significantly differentially expressed genes distinguishing obese women from overweight women is provided in Table S1 provided in the supplementary data to this report.

### 3.3. Association of TNBC with Obesity and Overweight in Postmenopausal Women

To investigate the association between TNBC and obesity or overweight in postmenopausal women, we compared gene expression levels of each patient group to patients with normal weight. Comparison of gene expression levels between individuals with normal weight and obese patients revealed a signature of 1556 significantly (*p* < 0.05) differentially expressed genes. The signature included 401 highly significantly (*p* < 0.01) differentially expressed genes. A signature of the top 44 most highly (*p* < 0.001) significantly differentially expressed genes associating obesity with TNBC is presented in Table 2. A complete list of all the significantly differentially expressed genes between normal and obese patients is presented in Table S2 provided as supplementary data to this report.

Analysis comparing patients with normal weight to overweight individuals produced a signature of 1327 significantly (*p* < 0.05) differentially expressed genes, of which 560 genes were highly significantly (*p* < 0.01) differentially expressed. A signature of the top 44 most highly significantly (*p* < 0.001) differentially expressed genes are presented in Table 2. A complete list of significantly differentially expressed genes distinguishing women with normal weight from women with overweight is presented in Table S2 provided as supplementary data to this report. There was a small overlap between genes associating obesity with TNBC and genes associating overweight status with TNBC.

To address the hypothesis that molecular perturbation differs between overweight and obese postmenopausal women we compared gene expression levels between the two patient groups. The analysis revealed a signature of 1438 significantly (*p* < 0.05) differentially expressed genes. The signature included 367 highly significantly (*p* < 0.01) differentially expressed genes. The most highly significantly (*p* < 0.001) differentially expressed genes were *ZNF230*, *PANX1*, *KLF9*, *EHD4*, *ACOT11*, *SPPL3*, *SEC23A*, *SEC62*, *CSGALNACT1*, *CCNY*, *WWC2*, *SNX19*, *WBP1L*, *COPS8*, *PPP2R2A*, *LRRFIP1*, *SMG7*, *ARF1*, *DUSP4*, *LOC101927523*, *LRCH3*, *BCAP29*, *PDPN*, *SMS*, *TRPC1*, *ANGPT2*, *ZNF140*, *PKD2*, *PLP2*, *CCDC7*, *SSBP2*, *CYP2U1*, *MGAT2*, *FOXP2*, *YWHAZ*, *IGBP1*, *STK17B*, *KCNQ3*, *DUSP1*, *TRIM32*, *SCARB1*, *PTGER4*, *PICALM*, *PSMF1*, and *JRK*. A complete list of significantly differentially expressed genes distinguishing overweight from obese individuals in postmenopausal women is presented in Table S2 provided as supplementary data to this report.

### 3.4. Premenopausal Versus Postmenopausal

**Figure 2:**
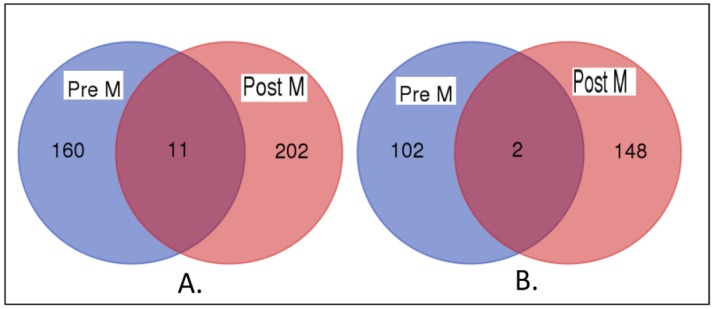
Venn diagrams showing overlap and lack thereof between premenopausal and postmenopausal women for genes associating (**A**) obesity or (**B**) overweight status with TNBC. Pre M and Post M denote premenopausal and postmenopausal, respectively.

A critical knowledge gap is whether the molecular mechanisms associating obesity or overweight in premenopausal women are the same mechanisms in postmenopausal women. To address this question, we evaluated the genes associating either obesity or overweight with TNBC in the two patient groups. We sought to discover genes associating obesity or overweight with TNBC, which overlap between the two groups and genes specific to each patient group. The results showing genes which overlap or do not overlap between premenopausal women and postmenopausal women are presented in Venn diagrams in Figure 2 (2A for obese women and 2B for overweight women).

There was little overlap between the two patient groups. Among the genes associating obesity with TNBC in premenopausal and postmenopausal women that were evaluated, 11 genes overlapped between the two patient groups, 202 were specific to postmenopausal women, and 160 genes were specific to premenopausal women (Figure 2A). Among the genes associating overweight status with TNBC in the two groups of women, only 2 genes overlapped, 148 genes were specific to postmenopausal women, and 102 genes were specific to premenopausal women (Figure 2B). This suggests that obesity could potentially have different effects on risk of TNBC in premenopausal and postmenopausal women.

### 3.5. Similarity in Patterns of Gene Expression Profiles

To investigate whether genes associating overweight or obesity with TNBC are co-regulated and have similar patterns of expression profiles, we performed unsupervised analysis using hierarchical clustering by menopausal status. For this analysis, we focused on genes strongly (*p* < 0.001) associating obesity or overweight with TNBC to minimize spuriousness in the patterns of expression profiles.

**Figure 3:**
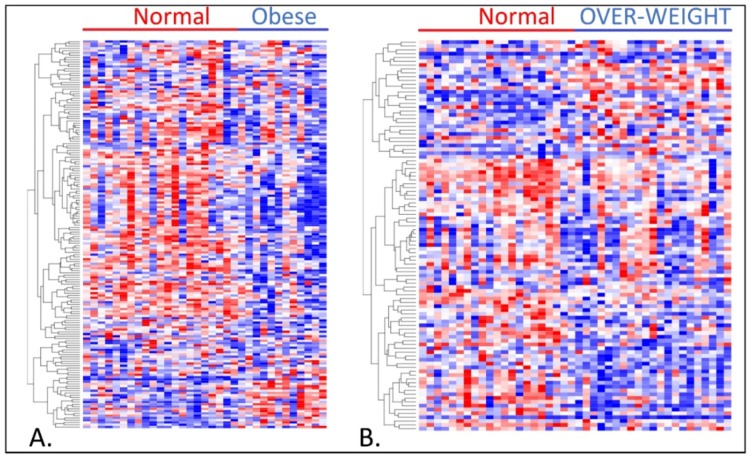
(**A**) Patterns of gene expression profiles for the 171 genes associating obesity with TNBC in premenopausal women. (**B**) Patterns of gene expression profiles for the 102 genes associating overweight status with TNBC premenopausal women. Genes in rows and patients in columns. Red color indicates upregulated and blue color indicates downregulated.

**Figure 4:**
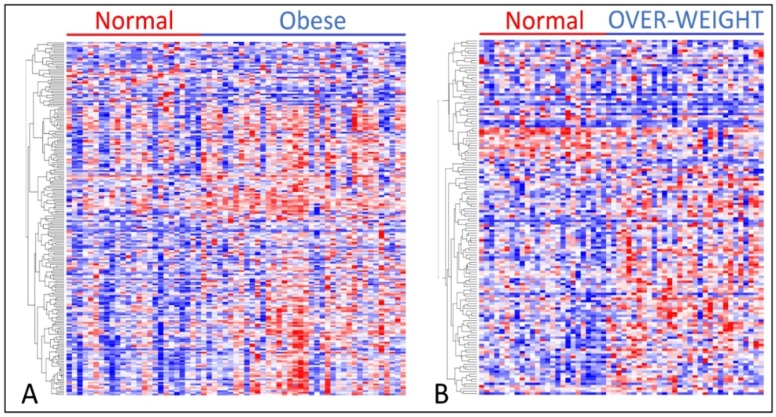
(**A**) Patterns of gene expression profiles for the 213 genes associating obesity with TNBC in postmenopausal women. (**B**) Patterns of gene expression profiles for the 146 genes associating overweight with TNBC in postmenopausal women. Genes in rows and patients in columns. Red color indicates upregulated and blue color indicates downregulated.

The results showing patterns of expression profiles for the 171 genes associating obesity with TNBC for premenopausal women are presented in Figure 3A. The results for the 102 genes associating overweight status with TNBC for premenopausal women are presented in Figure 3B. Figure 4A,B show the patterns of expression profiles for the 213 genes associating obesity with TNBC and the 146 genes associating overweight status with TNBC in postmenopausal women. In both premenopausal women (Figure 3A,B) and postmenopausal women (Figure 4A,B), the genes were co-expressed and had similar patterns of expression profiles. As expected, there were significant variations in patterns of expression profiles. The spuriousness in patterns of gene expression profiles could be explained partially by the heterogeneity in the patient samples. TNBC is inherently a heterogeneous disease consisting of different subtypes, and thus under such conditions the observed outcome was expected.

### 3.6. Molecular Networks and Biological Pathways Associating Obesity with TNBC

To gain insights about the broader biological context in which genes associating obesity with TNBC operate in premenopausal and postmenopausal women, we performed network and pathway analysis by menopausal status using IPA. We hypothesized that genes associating obesity with TNBC are functionally related and interact with one another in molecular networks and biological pathways. We sought to discover molecular networks and biological pathways associating obesity with TNBC. Only the genes that highly significantly (*p* < 0.01) associated obesity with TNBC in premenopausal and postmenopausal women were used in this analysis.

**Figure 5:**
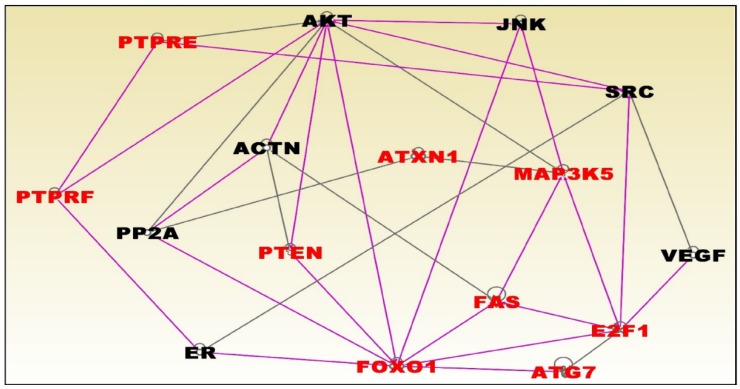
Molecular networks containing genes predicted to be significantly associated with obesity in premenopausal women. Network analysis was based on highly significantly differentially expressed genes (*p* < 0.01) in red fonts. Gene symbols in red fonts were predicted to be highly significantly associated with obesity. Genes in black symbols are predicted to be functionally related to genes in red fonts. The pink and black lines denote the relationships between merged networks.

**Figure 6:**
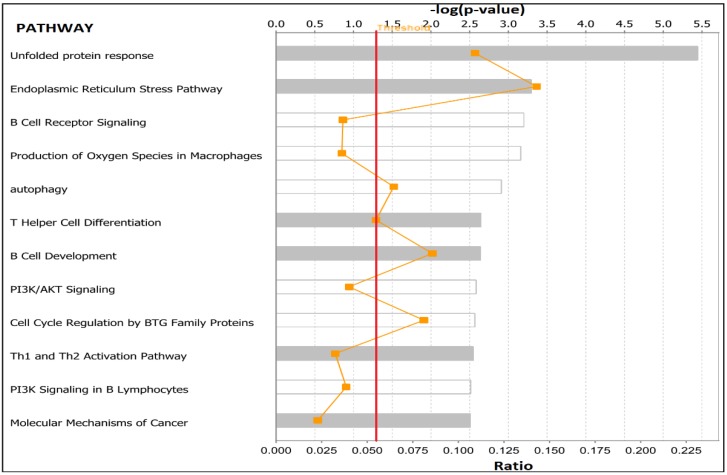
Biological pathways predicted to highly significantly associate obesity with TNBC in premenopausal women. The red line indicates the threshold level above which significance is declared. The zigzagging orange line denotes the ratio of the number of genes predicted to map to that pathway to the original number of genes in that pathway.

The results of network and pathway analysis for premenopausal women are presented in Figure 5 and Figure 6; respectively. Network analysis revealed genes predicted to be significantly involved in cell cycle, cell death and survival, cellular development, cellular growth and proliferation, cell morphology, and cellular function and maintenance. The most significant genes in the network included *PTPRF*, *E2F1*, and *ATG7* which were upregulated and the genes *PTPRE*, *PTEN*, *ATXN1*, *MAP3K5*, *FAS* and *FOXO1* were downregulated (Figure 5).

Pathway analysis revealed biological pathways highly significantly associated with unfolded protein response, endoplasmic reticulum stress pathway, the B cell receptor signaling pathway, production of nitric oxide and reactive oxygen species in macrophages and the autophagy signaling pathways (Figure 6). The top upstream regulator genes discovered by pathway analysis included *CD3*, *SEL1L*, *TGFB1*, and *TNFSF11*.

**Figure 7:**
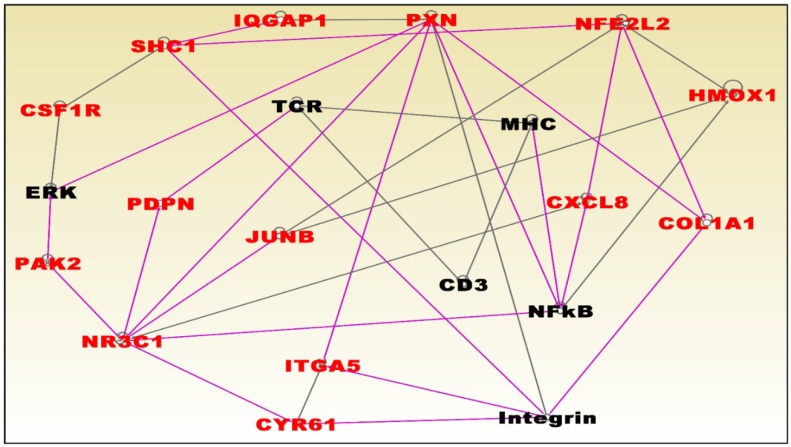
Molecular networks predicted to significantly associate obesity with TNBC in postmenopausal women. Gene symbols in red fonts were predicted to be highly significantly associated with obesity. Genes in black symbols are predicted to be functionally related.

**Figure 8:**
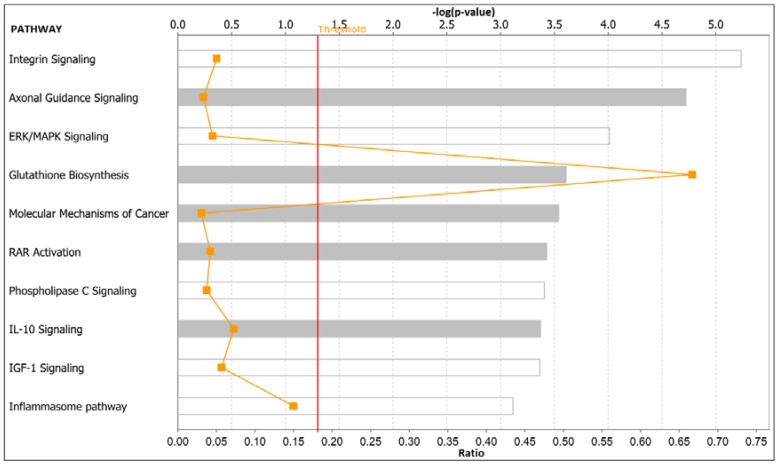
Top biological pathways predicted to be highly significantly associating obesity with TNBC in postmenopausal women. Pathway analysis was based on the most significantly differentially expressed genes. The red line indicates the threshold level above which significance is declared.

To discover molecular networks and biological pathways associating obesity with TNBC in postmenopausal women, we performed additional analysis as described in the preceding section. The results of network and pathway analysis are presented in Figure 7 and Figure 8, respectively. Network analysis revealed molecular networks containing genes predicted to be significantly involved in cellular movement, cell-to-cell signaling and interactions, cell death and survival, cellular function and maintenance, cell development, drug metabolism, and cellular growth and proliferation (Figure 7). The most significant genes in the networks were *HMOX1* which was upregulated and the genes *CSF1R*, *SHC1*, *IQGAP1*, *PXN*, *CXCL8*, *COL1A1*, *ITGA5*, *CYRG1*, *JUNB*, *PDPN*, *PAK2*, and *NR3C1*, which were downregulated (Figure 7). Pathway analysis revealed the integrin, axonal guidance, hepatic fibrosis, extracellular signal-regulated kinase (ERK)/mitogen-activated protein kinase (MAPK) (ERK/MAPK), and signaling pathways predicted glutathione biosynthesis signaling pathways (Figure 8). In addition, pathway analysis revealed the upstream regulators including *TNF*, *TGFB1*, cycloheximide, lipopolysaccharide, and *IL1*.

## 4. Discussion

Obesity and overweight have been reported to be correlated with an increased risk of developing TNBC in epidemiologic studies [8,9]. However, the epidemiologic studies that have associated overweight status and/or obesity with TNBC have yielded mixed results [17,18,19]. Most notably, there is little information in the published literature about the molecular markers associating overweight and or obese status with TNBC in premenopausal and postmenopausal women. In an effort to begin to address this knowledge gap, we conducted this exploratory investigation using publicly available gene expression data to elucidate the potential relationship between TNBC and obesity or overweight status in premenopausal and postmenopausal women. The investigation revealed that in both premenopausal and postmenopausal women, obesity and overweight were associated with TNBC. This suggests that overweight and obesity are likely to play a role in the etiology of TNBC. These results are consistent with several epidemiological studies which have associated obesity or overweight status with TNBC [8,9]. The novel aspect of this study is that it delineates the molecular mechanisms associating overweight and obesity with TNBC in both premenopausal and postmenopausal women. To our knowledge this is the first study to use transcription profiling to investigate the association between obesity and TNBC in both premenopausal and postmenopausal women. Two recent studies have associated obesity with breast cancer, primarily of the ER-positive type [35,36]. Our analysis focused on the TNBC type, the most aggressive form of breast cancer, and examined both pre and postmenopausal women.

The clinical significance of the results in this study is that, given the expanding obesity epidemic in the United States and the lack of targeted therapies for TNBC, the discovered biomarkers (if confirmed) could be used for precision prevention and the development of novel therapeutics. Although our study did not address the relationship between obesity and clinical outcomes due to lack of such information in the data set we used, previous epidemiological studies have shown that overweight status is an independent prognostic factor for overall survival and disease-free survival [10,37].

In this study, there was a small overlap in genes associating obesity or overweight status with TNBC between premenopausal and postmenopausal women. This tends to suggest that obesity or overweight status may have different effects on premenopausal and postmenopausal women. This finding is consistent with the results reported in a recent dose-response meta-analysis report involving 3,318,796 subjects from 31 cohort studies, which showed that BMI had different effects on premenopausal and postmenopausal breast cancer risk [38]. The main difference between that study and ours is that the reported study did not use transcriptome data and did not focus on TNBC as ours does.

The discovery of different molecular networks and biological pathways associating obesity with TNBC in premenopausal and postmenopausal women suggests that the mechanisms of regulation may be different in the two groups of women. The clinical significance is that different pathways may be targeted in the two patient groups. The association of obesity with IL-10 (Interleukin-10) and the inflammasome pathways is very interesting, because previous studies have suggested that molecular perturbation in obese individuals with TNBC may be related to metabolism and inflammation [39,40]. Although we did not investigate this relationship in our study owing to lack of such information in our data set, previous epidemiologic studies have shown that before menopause, triple-negative breast cancers were related to obesity and chronic inflammation, and that after menopause, in women aged <65 the latter subtypes were related to metabolic syndrome [22,39,40,41,42].

The association of overweight with TNBC is particularly interesting because epidemiological studies have shown that overweight premenopausal women are at greater risk of death and progression than women with normal weight [10]. To the extent that this study was focused on women of European ancestry, the results are consistent with recent reports of epidemiological studies in Caucasian women [43]. For example, a recent epidemiology study on obesity and TNBC involving socio-economically deprived Caucasian women in the Appalachian in West Virginia revealed the occurrence of TNBC in younger women with a later stage of diagnosis [43].

**Limitation of the study:** Although this exploratory study provides some insights, when associating obesity and overweight status with TNBC in premenopausal and postmenopausal women, some limitations must be acknowledged. We used publicly available data which has several limitations including a small sample size, lack of outcome information such as survival, and heavy reliance on Caucasian women, which severely limits the scope and generalization of the results of the study. Given the heterogeneity inherent in TNBC, the sampling errors that could potentially emanate from small sample size, and the recognition that gene expressions can be TNBC subtype-specific, we view this study as an exploratory investigation and recommend that further studies using independent cohorts are warranted in this line of research.

Although our study did not include African American women, previous epidemiologic studies have reported that the incidences and mortality rates in patients diagnosed with TNBC are significantly higher in African American (AA) women and that the disease tends to have a higher impact in premenopausal AA women regardless of age or BMI [2,44,45]. However published reports on survival outcomes for African-American women with TNBC relative to European-American women are conflicting [2,46,47]. Therefore, although we did not use the AA women in this study, the significance of this exploratory investigation is that both obesity and overweight are modifiable risk factors affecting both AA women and women of European ancestry (EA) diagnosed with TNBC [44,45,48] and therefore warrant further investigations in a cohort involving both ethnic populations. Indeed, the reliability of the results could have significantly improved if we replicated the study using a data set from an independent cohort. However, the lack of availability of a data set with similar characteristics limited our ability to address that issue. This is a weakness inherent in the use of publicly available data that we readily acknowledge but is beyond the scope of this report. While our study did not address individual subtypes of TNBC, a previous epidemiologic study [49] has reported the correlation between BMI and breast cancer subtypes, suggesting that further investigation in this line of research may be warranted. However, such a study would require a much larger sample size, which was the rate limiting factor in this investigation. Lastly, in this study we used BMI as the surrogate measure of overweight status and obesity. It is conceivable that other biological mechanisms which we did not consider in this study (such as adiposity) may be more reliable measures of obesity than BMI [50] and are worth exploring. In this study we considered BMI as the surrogate measure of both obesity and overweight status because consistent with the WHO and the CDC guidelines [24,25], it is a simple, inexpensive, and noninvasive surrogate measure of body fat [24]. In contrast to other methods, BMI relies solely on height and weight. Therefore, with access to the proper equipment, individuals can have their BMI routinely measured and calculated with reasonable accuracy. Importantly, studies have shown that BMI levels correlate with body fat and with future health risks [24,25]. Thus, to the extent that high BMI is a good predictor of future morbidity and death in TNBC [37], it is an appropriate measure for screening for obesity and its health risks in TNBC patients.

## 5. Conclusions

The results of this exploratory study show that overweight and obese status are associated with TNBC in premenopausal and postmenopausal Caucasian women. The results further demonstrate that obesity and overweight status could potentially have divergent impacts in premenopausal and postmenopausal women. More research involving larger sample sizes from different races/ethnic populations is needed to confirm these results.

## Figures and Tables

**Table 1:** Top 32 most highly significantly differentially expressed genes associating overweight and or obese status with triple-negative breast cancer (TNBC) in premenopausal women.

Normal Weight Versus Overweight			Normal Weight Versus Obese		
Gene Symbol	Cytoband	*p*-Value	Gene Symbol	Cytoband	*p*-Value
*COX10*	17p12	0.000166	*DUXAP8*	22q11.1	0.0000984
*DPY19L1*	7p14.2	0.000236	*ABCG1*	21q22.3	0.000115
*MYL6B*	12q13.2	0.000242	*IFNGR1*	6q23.3	0.000187
*CD84*	1q23.3	0.000321	*TPST2*	22q12.1	0.000191
*RAPGEF1*	9q34.13	0.000325	*CCDC32*	15q15.1	0.000205
*PAWR*	12q21.2	0.000396	*SFT2D1*	6q27	0.00024
*RPS21*	20q13.33	0.000399	*SMC2*	9q31.1	0.00024
*IL13RA1*	Xq24	0.000496	*NPC2*	14q24.3	0.000251
*GABBR1*	6p22.1	0.000506	*MGAT4A*	2q11.2	0.000256
*SLF2*	10q24.31	0.000514	*RNASET2*	6q27	0.000261
*ZNF621*	3p22.1	0.000586	*WAPL*	10q23.2	0.000261
*TOP1MT*	8q24.3	0.000644	*ATXN1*	6p22.3	0.000267
*MMD2*	7p22.1	0.00068	*ALG5*	13q13.3	0.00027
*N4BP2L2*	13q13.1	0.000742	*PTEN*	10q23.31	0.000358
*SNX29*	16p13.13	0.000777	*CREBL2*	12p13.1	0.000429
*CCDC34*	11p14.1	0.000803	*SUSD6*	14q24.1	0.000447
*EPB41L4A*	5q22.1	0.000829	*ZC3H7B*	22q13.2	0.000461
*MAP3K3*	17q23.3	0.000887	*ITFG1*	16q12.1	0.000497
*LYST*	1q42.3	0.00093	*FXN*	9q21.11	0.000527
*STC2*	5q35.2	0.001075	*BLVRA*	7p13	0.000542
*IQGAP1*	15q26.1	0.001177	*ICAM3*	19p13.2	0.000554
*KYNU*	2q22.2	0.001258	*XBP1*	22q12.1	0.000671
*FBXO28*	1q42.11	0.001358	*CLN5*	13q22.3	0.000682
*ARSD*	Xp22.3	0.001402	*SEL1L*	14q31	0.000687
*NBN*	8q21.3	0.001535	*DNAJC3*	13q32.1	0.000716
*IFNGR1*	6q23.3	0.001573	*LAYN*	11q23.1	0.000735
*CASP9*	1p36.21	0.001591	*GPRIN3*	4q22.1	0.000833
*EHD4*	15q15.1	0.00167	*WIPF1*	2q31.1	0.000838
*SSH1*	12q24.11	0.001679	*SET*	9q34.11	0.000896
*SYN2*	3p25.2	0.001725	*FOXN3*	14q31.3	0.00093
*ZNF585A*	19q13.13	0.001861	*FBXO10*	9p13.2	0.000936
*FCGR3A*	1q23.	0.001938	*SNX29*	16p13.13	0.000962

**Table 2:** Top 44 most highly significantly differentially expressed genes associating obese and or overweight status with TNBC in postmenopausal women. NW = Normal weight.

Gene Symbol	NW vs. Obese		Gene Symbol	NW vs. Overweight	
	Cytoband	*p*-Value		Cytoband	*p*-Value
*MSX1*	4p16.2	0.000002	*TAGLN2*	1q23.2	0.0000002
*IL4R*	16p12.1	0.0000023	*ZNF92*	7q11.21	0.0000778
*STK10*	5q35.1	0.0000194	*KEAP1*	19p13.11	0.0000934
*PLK3*	1p34.1	0.0000257	*ZNF253*	19p13.11	0.0000988
*EPHA2*	1p36	0.0000278	*YWHAH*	22q12.3	0.000138
*MUT*	6p12.3	0.0000321	*PLEC*	8q24	0.000146
*SERPINE1*	7q22.1	0.0000412	*TMC6*	17q25.3	0.000161
*MVP*	16p11.2	0.0000842	*CARHSP1*	16p13.2	0.000196
*TNC*	9q33.1	0.000134	*YWHAZ*	8q22.3	0.000224
*OSMR*	5p13.1	0.000138	*JRK*	8q24.3	0.000255
*MESDC1*	15q25.1	0.000141	*PROM2*	2q11.1	0.000269
*RBMS1*	2q24.2	0.000196	*SMAD5*	5q31.1	0.000284
*PDPN*	1p36.21	0.000199	*ZNF138*	7q11.21	0.000293
*ZEB2*	2q22.3	0.000218	*MESDC1*	15q13	0.000314
*CTDSP2*	12q14.1	0.000243	*ZNF85*	19p12	0.000344
*PTPRE*	10q26.2	0.000304	*ZNF736*	7q11.21	0.00042
*TWIST2*	2q37.3	0.000335	*NADSYN1*	11q13.4	0.00048
*PML*	15q24.1	0.000349	*SRSF9*	12q24.31	0.000492
*C6orf141*	6p12.3	0.000386	*BAHD1*	15q15.1	0.000518
*WWTR1-AS1*	3q25.1	0.000441	*ZBTB3*	11q12.3	0.000528
*MGAT1*	5q35.3	0.000446	*SRSF7*	2p22.1	0.000554
*GCLC*	6p12.1	0.000463	*BBS9*	7p14.3	0.000619
*PELO*	5q11.2	0.000495	*FOSL2*	2p23.2	0.000661
*SLC2A3*	12p13.31	0.000506	*ACLY*	17q21.2	0.000676
*SSH1*	12q24.11	0.000542	*KANSL1L*	2q34	0.000728
*PRKACA*	19p13.1	0.000627	*TFAP2A-AS1*	6p24.3	0.000766
*PXN*	12q24.23	0.000652	*SMG7*	1q25.3	0.000771
*PLEC*	8q24	0.000673	*DUOXA1*	15q21.1	0.000772
*FAS*	10q23.31	0.000737	*HNF4A-AS1*	20q13.12	0.0008
*DUSP1*	5q35.1	0.000777	*EPHA2*	1p36.13	0.000811
*PTAFR*	1p35.3	0.000786	*C11orf54*	11q23.1	0.00097
*SCARF1*	17p13.3	0.000807	*ZNF506*	19p13.11	0.000979
*KLF7*	2q3.3	0.000839	*C11orf57*	11q23.1	0.001022
*FPR1*	19q13.41	0.000848	*HPCAL1*	2p25.1	0.001038
*CD163*	12p13.31	0.000862	*KLHDC7B*	22q13.33	0.001083
*RAP1B*	12q15	0.000879	*PLP2*	Xp11.23	0.001178
*CTSL*	9q21.33	0.000896	*ZNF592*	15q25.2	0.001202
*GGT5*	22q11.23	0.000929	*TTLL13P*	15q26.1	0.001204
*GLI2*	2q14.2	0.000951	*RAB34*	17q11.2	0.001223
*ITGA5*	12q13.13	0.00097	*ATM*	11q22.3	0.001286
*RBBP6*	16p12.1	0.000979	*STX8*	17p13	0.001314
*MKL1*	22q13.1	0.000982	*PAX8*	2q14.1	0.001328
*ST7L*	1p13.2	0.000986	*ODF3*	11p15.5	0.001342
*ANGPT2*	8p23.1	0.000992	*NEK1*	4q33	0.001358

## Supplementary Materials

The following are available online at http://www.mdpi.com/1660-4601/15/11/2338/s1: Table SA: List of significantly differentially expressed genes showing evidence of association with TNBC at *p* < 0.05 among the three patient groups in women with premenopausal status. Table SB: List of significantly differentially expressed genes showing evidence of association with TNBC at *p* < 0.05 among the three patient groups in women with postmenopausal status. Table S1: List of significantly differentially expressed genes showing evidence of association between TNBC and obesity or overweight status in women with premenopausal status. Also shown in this table is a list of significantly differentially expressed genes distinguishing obese from non-obese women. Table S2: List of significantly differentially expressed genes showing evidence of the association between TNBC and obesity or overweight status in women with postmenopausal status. Also shown in this table is a list of significantly differentially expressed genes distinguishing obese from non-obese women.
